# A chromosomal-level reference genome of the widely utilized *Coccidioides posadasii* laboratory strain “Silveira”

**DOI:** 10.1093/g3journal/jkac031

**Published:** 2022-02-07

**Authors:** Marcus de Melo Teixeira, Jason E Stajich, Jason W Sahl, George R Thompson, Rachel B Brem, Claire A Dubin, Austin V Blackmon, Heather L Mead, Paul Keim, Bridget M Barker

**Affiliations:** Faculty of Medicine, University of Brasília, Brasília 70910-900, Brazil; The Pathogen and Microbiome Institute, Northern Arizona University, Flagstaff, AZ 86011, USA; Institute for Integrative Genome Biology, University of California Riverside, Riverside, CA 92521, USA; Department of Microbiology and Plant Pathology, University of California Riverside, Riverside, CA 92521, USA; The Pathogen and Microbiome Institute, Northern Arizona University, Flagstaff, AZ 86011, USA; Department of Medical Microbiology and Immunology, University of California Davis, Davis, CA 95616, USA; Department of Plant and Microbial Biology, University of California Berkeley, Berkeley, CA 94720, USA; Department of Plant and Microbial Biology, University of California Berkeley, Berkeley, CA 94720, USA; The Pathogen and Microbiome Institute, Northern Arizona University, Flagstaff, AZ 86011, USA; The Pathogen and Microbiome Institute, Northern Arizona University, Flagstaff, AZ 86011, USA; The Pathogen and Microbiome Institute, Northern Arizona University, Flagstaff, AZ 86011, USA; The Pathogen and Microbiome Institute, Northern Arizona University, Flagstaff, AZ 86011, USA

**Keywords:** valley fever, long-read sequencing, funannotate, human fungal pathogen, fungal genomes, reference genome, coccidioidomycosis

## Abstract

Coccidioidomycosis is a common fungal disease that is endemic to arid and semi-arid regions of both American continents. *Coccidioides immitis* and *Coccidioides posadasii* are the etiological agents of the disease, also known as Valley Fever. For several decades, the *C. posadasii* strain Silveira has been used widely in vaccine studies, is the source strain for production of diagnostic antigens, and is a widely used experimental strain for functional studies. In 2009, the genome was sequenced using Sanger sequencing technology, and a draft assembly and annotation were made available. In this study, the genome of the Silveira strain was sequenced using single molecule real-time sequencing PacBio technology, assembled into chromosomal-level contigs, genotyped, and the genome was reannotated using sophisticated and curated in silico tools. This high-quality genome sequencing effort has improved our understanding of chromosomal structure, gene set annotation, and lays the groundwork for identification of structural variants (e.g. transversions, translocations, and copy number variants), assessment of gene gain and loss, and comparison of transposable elements in future phylogenetic and population genomics studies.

## Introduction

Coccidioidomycosis is a multisymptomatic mycotic disease affecting humans and other animals in arid and semi-arid regions in the Americas (Van Dyke *et al.* 2019). When aerosolized, conidia can be inhaled into the lungs of a susceptible host. In humans, the infection is asymptomatically controlled in 60% of infections (Nguyen *et al.* 2013). However, the infection can progress from a mild pneumonia to a severe acute or chronic pulmonary infection. Moreover, the disease can disseminate into multiple organs (e.g. bones, skin, spleen, etc.) including the meninges, which is often fatal without treatment, and with the potential necessity of lifelong antifungal therapy (Galgiani *et al.* 2020). *Coccidioides immitis* and *Coccidioides* *posadasii* diverged around 5.1 MYA (Engelthaler *et al.* 2016) and are able to successfully interbreed, as evidenced by hybrids and patterns of introgression (Neafsey *et al.* 2010; Maxwell *et al.* 2019). Population structure of both species has been defined in several studies and generally reveals biogeographic patterns of distribution across North and South America (Fisher *et al.* 2001; Teixeira *et al.* 2019). *Coccidioides* *immitis* is composed of up to 3 populations: San Joaquin (Central) Valley, San Diego/Mexico, and Washington, while 3 populations have been described within *C. posadasii*: Arizona, Texas/Mexico/South America and Caribbean (Teixeira *et al.* 2019).

The *C. posadasii* Silveira strain was collected from a patient of Dr. Charles E. Smith in 1951 (Friedman *et al.* 1955, 1956). The patient had severe primary coccidioidomycosis with erythema nodosum, but recovered after several months of illness. Extensive testing in mice revealed that the strain was highly infective via the intraperitoneal route and caused nearly 100% lethal infections with as few as 100 conidia by 90 days (Friedman *et al.* 1956). Early studies analyzing the genetic diversity of *Coccidioides* via restriction fragment length polymorphisms (RFLPs) and multilocus sequencing typing (MLST) of nuclear genes indicated that this strain grouped with the “non-California” population (Koufopanou *et al.* 1997). Although this strain was recovered from a patient residing in California at the time of diagnosis (prevalent area of *C. immitis*), subsequent genetic analysis demonstrated that this strain is *C. posadasii* (Fisher *et al.* 2002). Interestingly, the patient was a migrant farmworker that had previous travel history in Arizona in the year prior to diagnosis (chart review by GRT).

Despite the fact that this strain has been maintained in culture since the 1950s, many studies on virulence, vaccination challenge, and host response have been conducted without a loss of virulence in many labs. Mice challenged intranasally with 27 Silveira arthroconidia succumbed to infection rapidly, and the strain had an intermediate to high virulence when compared to other *C. immitis* and *C. posadasii* strains (Cox and Magee 2004). Silveira is able to initiate parasitic phase growth in vitro in Converse (Scalarone and Levine 1969) or RPMI media (Mead *et al.* 2019b), and early antigen preparations (coccidioidin and spherulin) for both intradermal cellular hypersensitivity and serologic testing were derived from this strain (Smith *et al.* 1961). Persistent skin reactivity was observed in people who recovered from primary coccidioidomycosis, which suggested that previous exposure to this fungus led to cellular immunity protection (Catanzaro *et al.* 1975). Early studies with this strain were seminal for correlating persistent protection to subsequent fungal exposure, and to the presence of viable *Coccidioides* cells in granulomas (Pappagianis *et al.* 1965; Pappagianis 1967). This strain was also used to demonstrate that hypersensitivity to coccidioidin in mice is mediated by cellular immunity and predicts protection against further infection (Cox *et al.* 1988).

Several immunization studies have been completed in various animal models using strain Silveira (Converse *et al.* 1962, 1964; Huppert *et al.* 1967; Peng *et al.* 1999; Hung *et al.* 2000). An auxotrophic *C. posadasii* Silveira mutant generated by cobalt-60 irradiation was attenuated for virulence in murine models and showed protective capabilities. However, in the case of the auxotrophic mutant, virulence was restored over time (Pappagianis *et al.* 1961; Walch and Kalvoda 1971). Inactivated cells and proteins from Silveira have been used for vaccination as well (Cox and Magee 2004). Mice immunized with formaldehyde-killed spherules, whole cell wall components, or proteins from Silveira showed a protective effect, even after challenge with different *Coccidioides* strains (Huppert *et al.* 1967; Zimmermann *et al.* 1998; Peng *et al.* 1999). The nucleotide and protein sequences of Silveira have been further characterized and used for the production of recombinant proteins with the aim of developing an effective vaccine (Peng *et al.* 1999; Shubitz *et al.* 2002).

The Silveira strain was deposited at 3 fungal repositories. One was by M.S. Collins in the 1970s, from the lab of Dr. Pappagianis to the American Type Culture Collection (ATCC 28868) and the other from Dr. M.A Brandt (CDC) also originally from Dr. Pappagianis’ lab (but via Dr. R. Cox lab) to the Westerdijk Fungal Biodiversity Institute (CBS 113859). Silveira is Lufenuron-resistant, and its growth is inhibited by polymyxin B and ambruticin (https://www.atcc.org/en/Products/All/28868.aspx). Presumably, each strain has been passaged both on plates and through mice multiple times over 70 years, but the exact accounting for each research group is unknown. Based on microsatellite profiles of strains from Dr. R. Cox and Dr. D. Pappagianis’ labs, there are 1 and 2 nucleotide length differences observed at 2 loci, K01 and K09 (Fisher *et al.* 2002). More recently, the strain was deposited by Dr. B.M. Barker to BEI resources (NR-48944), which was received from the lab of Dr. J.N. Galgiani, initially a gift from Dr. H. Levine, and this is the strain we sequenced in this study. Interestingly, this strain was genotyped as *C. posadasii*, ARIZONA population and the microsatellite genotype is further diverged from published data at 2 additional loci: GA37 and K07 (Fisher *et al.* 2002; Teixeira and Barker 2016).

The genome of the NR-48944 Silveira strain was previously sequenced using Sanger-capillary technologies to a 5.2x coverage, which was assembled into a 27.58 Mbp genome containing 54 nuclear scaffolds and 3 mitochondrial scaffolds with 10,228 protein-coding genes annotated (Neafsey *et al.* 2010). Recently, the proteins from lysates and filtrates of both filamentous and parasitic phases of Silveira were sequenced using a GeLC‐MS/MS approach (Mitchell *et al.* 2018). The authors reported 9,024 peptides from 734 previously annotated proteins, with 103 novel proteins described.

Previous sequencing efforts resulted in an unclosed draft genome and draft annotation (Neafsey *et al.* 2010). We therefore finished and reannotated the genome to produce an updated assembly and annotation resource. A combination of both long-read single molecule real-time sequencing (SMRT) PacBio and paired short-read Illumina MiSeq technology was used to produce a chromosomal-level high-quality assembly. Our assembly approach resulted in 5 complete chromosomes, and a complete mitochondrial genome. The new annotation pipeline utilized a combination of transcriptomic and proteomic evidence, and generated a reduced number of total annotated genes compared to the original draft annotation.

## Materials and methods

### Strains and public data

We sequenced the *C.* *posadasii* strain Silveira that we obtained from the J.N. Galgiani collection, who originally received the strain from H. Levine, and we deposited this strain at BEI Resources (NR-48944). This strain has been deposited by other researchers previously to ATCC and Westerdijk Institute/CBS-KNAW resources (ATCC 28868 and CBS 113859). The first Sanger-based genome assembly of *C.* *posadasii* strain Silveira was downloaded from the NCBI (https://www.ncbi.nlm.nih.gov/nuccore/294654294) for comparisons, and the DNA used for that sequencing was from the J.N. Galgiani lab, albeit an earlier passage than ours. In addition, the genome data from *C.* *immitis* strains RS and WA_211 were retrieved from NCBI (https://www.ncbi.nlm.nih.gov/nuccore/AAEC00000000 v3, https://www.ncbi.nlm.nih.gov/nuccore/1695747985). The annotation for RS was last updated in March of 2015.

### DNA extraction

DNA extraction for short and long-read sequencing was initiated by growing the Silveira strain from arthroconidia in liquid 2xGYE (2% glucose, 1% Difco Yeast Extract in dddH_2_O) for 120 h and harvesting by centrifugation and washing twice with sterile 1xPBS. DNA for the short-read library was obtained by lysing cells in SDS lysis buffer with bead-beating and using a phenol: chloroform: isoamyl alcohol (25:24:1, v/v) with isopropanol precipitation method (Mead *et al.* 2019a). DNA for the long-read library was obtained by freezing mycelia in liquid nitrogen and grinding in a sterile mortar and pestle to a fine powder in a biological safety cabinet in a biosafety level 3 laboratory following the U’Ren high molecular weight (HMW) fungal DNA laboratory protocol (U’Ren and Moore 2018). Briefly, approximately 3 g of ground fungal biomass was added to 14 ml SDS buffer (20 mM Tris-HCl pH 7.5, 1 mM EDTA pH 8, 0.5% w/v SDS) and incubated at 65°C, followed by addition of 0.5X potassium-acetate (5M KOAc, pH 7.5) to SDS buffer and incubation on ice for 30 min. The sample was centrifuged and the supernatant was subjected to isopropanol precipitation and ethanol cleanup. The resulting pellet was suspended in TE buffer (Tris-HCl 1 mM, EDTA 0.5 mM) and RNase treated, followed by phenol/chloroform extraction and ethanol precipitation. The final DNA pellet was slowly suspended in a minimal volume of low salt TE for sequencing. HMW DNA was quality checked for size using chromatin electrophoresis gel, for purity on Nanodrop 2000 (Thermo Fisher, USA) for 260/280 and 260/230 ratios, and quantified by Qubit (Invitrogen, USA) fluorometry.

### Sequencing

Long-read sequencing was performed at the University of Arizona Genomics, Tucson, Arizona, sequencing core facility. Briefly, a sequencing library was constructed from 6 µg of HMW DNA following the PacBio (Pacific Biosciences, USA) protocol for use with the SMRTbell Express Template Prep Kit. The ligated library templates were size selected on Sage BluePippin instrument (Sage Science, USA) for selection of fragments of 17 Kbp and larger, which is appropriate given the predicted smallest chromosome is greater than 4 Mbp (Pan and Cole 1992). The final purified sequencing library yield was 980 ng with a final mode size of 38.3 Kbp as determined by Fragment Analyzer (Agilent Technologies, USA). The final library was bound with PacBio polymerase and sequencing primer v3 using manufacturers’ methods. One 1M v2 SMRT cell was loaded with a bound library at a loading concentration of 7 pmol/cell followed by a 10-h sequencing run on the PacBio Sequel Instrument at the University of Arizona, Tucson, AZ, resulting in ∼475x coverage. In addition, short-read sequencing was performed on a MiSeq (Illumina) instrument at The Translation Genomics Research Institute, Flagstaff, Arizona, using a MiSeq reagent kit v2 (300-cycles) on a high output mode (Teixeira *et al.* 2019). This ∼100X sequencing coverage with Illumina short reads was generated to support PacBio read correction and assembly polishing as needed.

### Assembly

The reference genome assembly pipeline was comprised of 5 steps: (1) PacBio reads were corrected using Illumina reads and the tool LoRDEC v 0.9 (Salmela and Rivals 2014); (2) Corrected PacBio reads were assembled with Canu software v 1.7.1 (Koren *et al.* 2017); (3) An additional round of assembly using Illumina short reads combined with the Canu PacBio scaffolds as trusted contigs in the assembler SPAdes v 3.13 (Bankevich *et al.* 2012); (4) 5 rounds of Pilon (v 1.24) corrections improved the genome assembly to further reduce nucleotide base error variants (Walker *et al.* 2014), and; (5**)** we further scaffolded the assemblies to existing *C.* *immitis* strain RS reference genome to compare synteny (Sharpton *et al.* 2009) with RaGOO v 1.1 (Alonge *et al.* 2019). We evaluated and compared assembly accuracy with the hybrid assembly method MaSuRCA v 3.3.0 (Zimin *et al.* 2013, 2017), which incorporates the high performance long-read assembler Flye v 2.5 and integrates short-read correction of reads and assembly into the pipeline (Kolmogorov *et al.* 2019). Assembly quality was assessed by using summary statistics, the number of complete alignments of conserved fungal proteins using BUSCO v 2.0 (Simao *et al.* 2015; Vanderlinde *et al.* 2019), RNASeq read mapping from existing libraries (Whiston *et al.* 2012), and utilizing existing and de novo annotations of *Coccidioides* genomes (Sharpton *et al.* 2009; Neafsey *et al.* 2010). Transposable elements (TEs) and low complexity DNA sequences were assessed using RepeatMasker v 4.1.1 (Smit 2020). Telomeric repeats were identified using the FindTelomers python script (https://github.com/JanaSperschneider/FindTelomeres). The quality of newly and previously assembled genomes was assessed using the QUAST v5 pipeline (Gurevich *et al.* 2013). The sequence composition of predicted 18S rRNA genes was performed with SSU-Align v 0.1.1 (eddylab.org/software/ssu-align/). The assembly of the mitochondrial DNA using Illumina reads was performed using the SPAdes Genome Assembler v3.14.0 (Bankevich *et al.* 2012) with a kmer sizes 61, 91, and 127 (de Melo Teixeira *et al.* 2021). Long-read mapping to the mitochondrial scaffolds was accomplished using the Minimap2 algorithm (Li 2018), Unicycler v0.4.8 (Wick *et al.* 2017), Flye v2.9 using only mitochondrial aligned reads, and Raven v1.3.0 (Vaser and Šikić 2021) to address ambiguities from the initial Canu and Flye assemblies, and read alignments were visualized using the Tablet software (Milne *et al.* 2013) and OGDRAW (Greiner *et al.* 2019). Dot Plot comparisons were performed using Gepard (Krumsiek *et al.* 2007) and MAFFT v 7 (Katoh and Standley 2013). In addressing the quality of our assembly, we noted 5 large scaffolds, which are consistent with the inferred chromosome count in the published Silveira karyotype (Pan and Cole 1992), plus 3 small scaffolds that represented a discrepancy with current knowledge. To test if these small scaffolds were duplicates from the main chromosomes, we used the Illumina reads from Silveira and mapped them using BWA-MEM v0.7.17 (Li and Durbin 2009) to the 5 large scaffolds of our preliminary assembly.

### Annotation

The Funannotate pipeline v 1.7.4 (Stajich and Palmer 2020) was used to automate ab initio gene predictor training using BRAKER1 (Hoff *et al.* 2016), Augustus v 3.3 (Stanke *et al.* 2006), and GeneMark-ET v 4.57 (Ter-Hovhannisyan *et al.* 2008). This pipeline generated de novo assembly of transcripts with Trinity v2.10 (Haas *et al.* 2013) to examine variation in exons used in isoforms, and aligned to the genome. This evidence was used as input data for EVidence Modeler (EVM) software v1.1.1 to generate a consensus set of predicted gene models (Haas *et al.* 2008). Gene models were filtered for length, spanning gaps and TEs using a *Coccidioides*-specific library of repetitive DNA to further clean the dataset (Kirkland *et al.* 2018). AntiSMASH 5.1.1 (fungismash) was used to identify biosynthetic gene clusters (Blin *et al.* 2019). The mitochondrial annotation was performed using the MFannot and RNAweasel pipelines available at https://megasun.bch.umontreal.ca/.

### Phylogenomic classification

To characterize the phylogenetic position of the Silveira strain, we first used the assembled genome as reference for Illumina read mapping and SNP variant calling utilizing NASP v1.0 (Sahl *et al.* 2016). We mapped 61 available genomes of *C. posadasii* against our 5 Silveira scaffolds using the Burrows-Wheeler Aligner (v0.7.7) tool (Li and Durbin 2009). SNPs were called using the GATK (v3.3.0) toolkit (McKenna *et al.* 2010) using previous NASP protocols developed by our group for *Coccidioides* genotyping (Teixeira *et al.* 2019). SNPs were called using the UnifiedGenotyper method and the parameter “het” was set to 0.0.1 We filtered SNPs using the following parameters: QD = 2.0 ‖ FS_filter = 60.0 ‖ MQ_filter = 30.0 ‖ MQ_Rank_Sum_filter = −12.5 ‖ Read_Pos_Rank_Sum_filter = −8. Duplicated SNPs identified by NUCmer (REF) as located within duplicated loci in the reference as well those with less than 10x coverage or with less than 90% variant allele calls were purged from the final dataset. A total of 258,470 SNPs were retrieved and submitted for unrooted phylogenetic analysis via maximum-likelihood method implemented in the IQTREE software v1.6.12 (Nguyen *et al.* 2015). The best-fit model was set according to Bayesian Information Criterion to TN+F+ASC+R6 and the phylogenetic signal was tested using both Shimodaira–Hasegawa approximate likelihood ratio test (SH-aLRT) and ultrafast bootstrap support (Anisimova and Gascuel 2006; Minh *et al.* 2013). The phylogenetic tree was visualized using the Figtree software (http://tree.bio.ed.ac.uk/software/figtree/). The population structure of *C. posadasii* and the proportion of admixture of the Silveira strain was inferred based on unlinked SNPs using the fastSTRUCTURE software (Raj *et al.* 2014). The best population scenario (or *K* value) was calculated using the fastSTRUCTURE choosek.py application based on the lowest marginal likelihood and cross-validation test. The proportion of the admixture of each individual was plotted using the Structure Plot v2.0 pipeline (Ramasamy *et al.* 2014).

## Results and discussion

### Sequencing and assembly

**Fig. 1.**
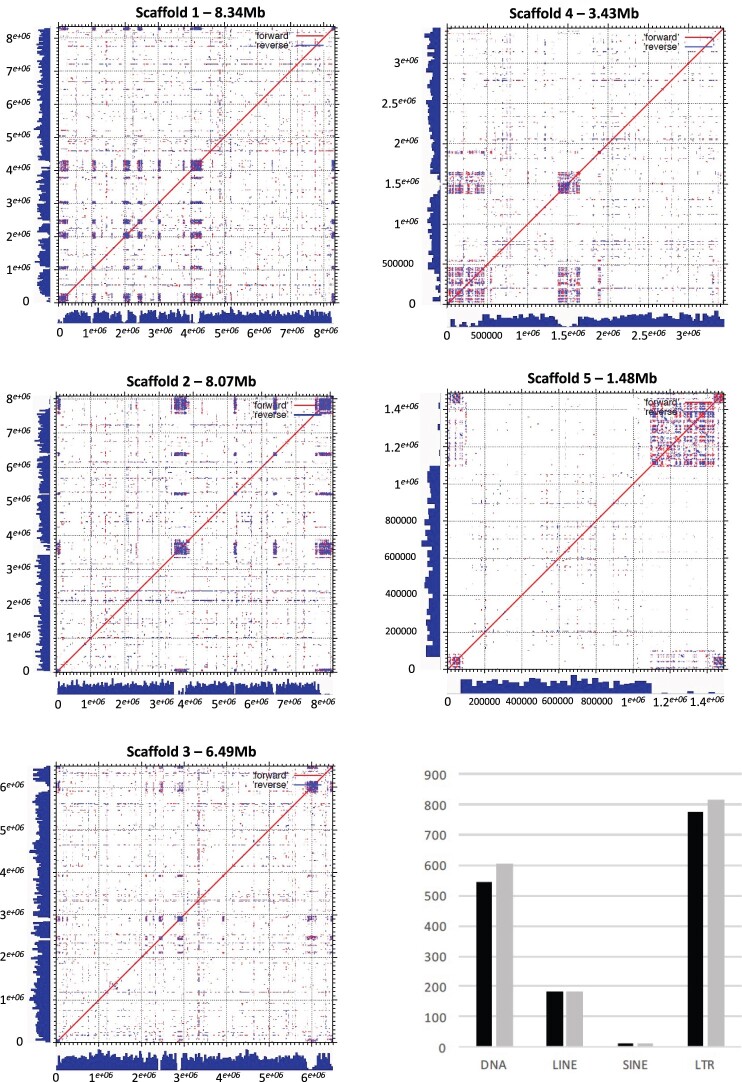
The gene density and repeats for Canu assemblies. Blue histograms on X/Y axes represent gene densities. Forward and reverse similarity blocks are indicated by red and blue dots, respectively. Central highly repeated sequence regions are the putative centromeres. Canonical telomeric repeats are indicated at both 5′ and 3′ ends. a) Dot Plot of Chromosome I. b) Dot Plot of Chromosome II. c) Dot Plot of Chromosome III. d) Dot Plot of Chromosome IV. e) Dot Plot of Chromosome V. f) Classes of repeats.

PacBio Sequel sequencing yielded ∼10.6 Gbp of raw data from over 643,000 reads with an average read length (N50) of ∼25 Kbp. Illumina MiSeq sequencing yielded ∼2.8 Gbp of raw data and >12 million paired-end short reads. We used both short-read Illumina and long-read PacBio data to assemble the genome using short-read-corrected (Pilon), long read (Canu), and a long-read alone (Flye) assembly approaches. The final genome was assembled with Canu on the corrected PacBio sequencing data into 9 scaffolds totaling 28.27 Mbp; scaffolds 1–5 representing chromosomes 1–5, scaffold 6 as the mtDNA, and scaffolds 7–9 are unassembled regions that we named contigs 6–8 (Table 1). The L50 metric for this assembly is 2 Mb, the N50 is 8.07 and the longest scaffold is 8.34 Mbp. Sequence searches for 18S rRNA genes identified 5 loci, 2 of them were complete with an additional that was nearly complete. By comparing the assembly metrics of the Silveira strain between Sanger and PacBio/Illumina assemblies, we observed a complete genome structure of this fungus consistent with its known chromosomal composition (Table 2, Supplementary Fig. 1) (Pan and Cole 1992). The genome size of the assembly using Canu is 690 Kbp longer than the previous Sanger assembly, which is contained in 54 nuclear genome scaffolds, with 3 mitochondrial scaffolds. The additional sequence length is likely due to improving assembly of repeated regions. The Canu assembly produced 5 large scaffolds which have 9 telomeric repeats (TTAGGG/CCCTAA)_n_ at both ends of scaffolds 1, 2, 3, and 5. On scaffold 4 this tandem repeat was only found at the forward strand in the Canu assembly (Fig. 1d). According to the densitometry analysis of pulsed-field gel electrophoresis (PFGE), the *C. posadasii* Silveira should have 4 chromosomal bands (Pan and Cole 1992). However, because scaffolds 1 and 2 are 8.34 and 8.07 Mbp, respectively, these minor chromosomal size differences would be difficult to discriminate using PGFE.

**Table 1.** Chromosome sizes.

Scaffold	COntig	Size (bp)
1	1	8,340,845
2	2	8,079,863
3	3	6,494,557
4	4	3,437,093
5	5	1,489,275
6	Mitochondrial genome	74,407
7	6	119,732
8	7	80,756
9	8	76,740

**Table 2.** Comparison of Silveira genomes.

	Silveira Sanger 2007	Silveira CANU 2020
Contigs	57	9
GC%	46.34	46.44
Predicted protein-coding genes	10,228	8,491
BUSCO completeness	95.3%	97.1%
Median gene length (bp)	2,109	1,810.5
Total number of introns	20,607	17,004
Total number of exons	32,511	26,348
Total number of CDS	30,840	26,216
tRNAs	119	111

### Chromosome structure and repetitive DNA

Highly-repeated DNA sequences play a pivotal role in genome architecture, evolution, and adaptation by modulating gene activity, and chromosomal rearrangements (Castanera *et al.* 2016; Sinha *et al.* 2017; Nieuwenhuis *et al.* 2019). The newly assembled Silveira genome has 17.12% total repetitive DNA assessed with RepeatMasker, while the previous version had 15.91%. The new assembly has 1,614 copies of various TEs, including SINEs, LINEs, LTR, and DNA transposons; the earlier version has 1,511. The most impacted TE classes were LTR retrotransposons and DNA transposons (Fig. 1f, Supplementary Table 1). This difference is likely due to improvements in the assembly because long-read technology can cover long stretches of repetitive DNA that may have been collapsed during the assembly process of Sanger and Illumina short reads. There is strong evidence for sub-telomeric repeats, as demonstrated by the presence of low complexity sequences adjacent to 9 terminal positions of the 5 larger scaffolds (Fig. 1). We did not observe telomeric repeats in the 3′ portion of the chromosome IV (Fig. 1d). We observed an accumulation of repetitive DNA and lack of protein-coding genes in central positions of the scaffolds, which may be centromeric repeats. We found that noncharacterized repeats were abundant in the putative centromeres; however, chromatin immunoprecipitation followed by deep sequencing are needed to determine whether those central repeats are in fact the centromeres of *C. posadasii*. Although we used multiple approaches to assemble genomes, the assemblies produced were largely in agreement, creating 5 large contigs (chromosomes), but since the canonical (TTAGGG/CCCTAA)_n_ eukaryotic repeats were only found in the Canu assembly, we used the Canu scaffolds for annotation and final assembly. In conclusion, for all 5 chromosomes, there were possible internal centromeric locations and canonical telomeres at the end except for 1 chromosome (Fig. 1).

### *Coccidioides immitis* genome comparison

A comparison of the assembled Silveira genome to the published RS (*C. immitis*) genome suggests that RS has slightly larger syntenic genome, with noted translocations and inversions (∼2 Mb, Supplementary Fig. 1c). The size difference could be due to sequencing technology and assembly methods, but may in fact represent a true difference between species, because we see a similar pattern when looking at *C. immitis* genome WA_211 (Supplementary Fig. c and d). Variation in genome size between 2 divergent species is possible, as well as differences among isolates of the same species. Alternatively, Silveira has been a laboratory isolate since the 1950s and has likely undergone micro-evolutionary changes due to in vitro selection for laboratory cultivation. Different isolates of the *Cryptococcus neoformans* var. *grubii* H99 genotype obtained from different laboratories display remarkable genetic variation related to microevolutionary processes of in vitro passage (Janbon *et al.* 2014). Genome reduction and other micro-evolutionary changes due to selective forces in the laboratory have been documented for other fungal pathogens (Chen *et al.* 2017; Ene *et al.* 2018). As high-fidelity long-read technologies become increasingly available, more accurate estimates of both genome size for *Coccidioides* spp. and structural genome variation among isolates will be possible.

**Fig. 2.**
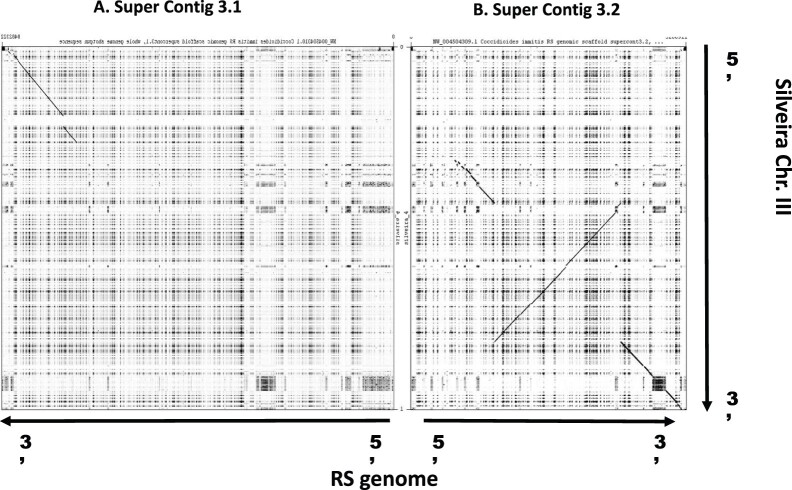
Silveira (*C. posadasii*) Chromosome III Dot Plot Similarity to the RS (*C. immitis*) Genome. The homologous regions of the Silveira chromosome III (6.5 mbp) are distributed across 2 super contigs of the RS genome assembly and not completely collinear. a) RS super contig 3.1 contains about 30% the 5′ Silveira chromosome III homologous sequences, with the remainder not homologous to chromosome III. Within the homologous region there a large gap of nonhomology. b) RS super contig 3.2 contains about 70% of Silveira chromosome III homologous sequences including a highly repetitive region at the Silveira 3′ end. The arrangement of the cross species homologous regions is not completely collinear with an apparent inversion of about half.

While most of the RS and Silveira genome assembles are syntenic (Supplementary Fig. 1), there is a notable exception for Silveira chromosome III (Fig. 2). Chromosome III aligns to the RS genome, but spans 2 RS contigs. RS supercontig 3.1 contains the 5’ 30% of Silveira chromosome III (∼3 Mb) with the remainder found on RS supercontig 3.2. The 3.2 supercontig alignment has a chromosomal rearrangement that is consistent with an inversion of ∼3 Mb. Genome assembly algorithms have difficulties in processing direct and indirect repeated sequences, and this can produce erroneous results (Tørresen *et al.* 2019). However, in this particular case, the inversion junction points are not associated with highly repetitive sequence. We manually examined the Silveira reads and assemblies around the junction points, and we found evidence that our chromosome III assembly is correct. Similar raw read data is not available for the RS genome, and these data are required to confirm this inversion. Chromosomal inversions and translocations can greatly influence meiotic processes by disrupting homolog pairing and recombination. Such reproductive barriers often exist between species and higher taxonomic relationships, limiting gene flow and allowing for differentiation and reinforcement of species boundaries. Again, the long in vitro culture history of Silveira leaves open the possibility that the apparent inversion may be a lab-specific or strain-specific genomic feature that will not be observed in all isolates of *C. posadasii*. Conversely, RS is also a lab strain that has been cultured under laboratory conditions since the 1950’s, with extensive evidence of hybridization and introgression, and thus the inversion could be specific for strain RS (Sharpton *et al.* 2009; Neafsey *et al.* 2010); however, we require additional long-read sequencing and assemblies to answer this specific question.

### Mitochondrial genome

**Fig. 3.**
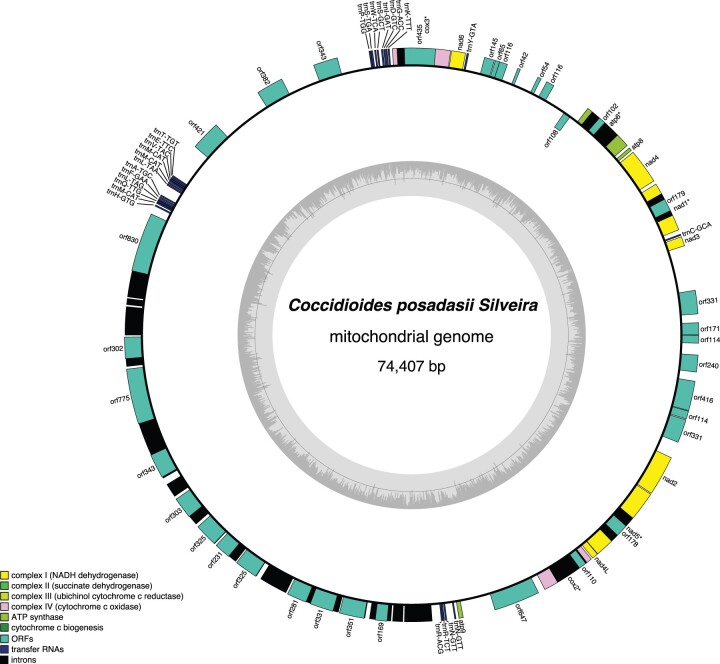
Final circular mitochondrial genome assembly of the *C. posadasii* Silveira strain. The circular plots show the core genes as part of the ubiquinone oxidoreductase, cytochrome b, cytochrome oxidase and ATP synthase protein complexes. Small and large ribosomal RNAs (*rns* and *rnl*), the RNAseP subunit (rnpB), as well as tRNAs are also displayed. Additional ORFs and introns are showed along the circular map.

Scaffold 6 corresponds to the mitochondrial genome that initially assembled to a size of 152 Kbp using Canu, which is almost double that of our previously published Illumina assembly version that suggested a 74 Kbp circular mapping genome (de Melo Teixeira *et al.* 2021). These results indicated assembly inconsistencies between the 2 sequencing technologies. The Flye assembly created by using reads only aligning to the mitochondrial sequence confirmed that the mitogenome was circular, but in fact was only 74 Kbp, which is consistent with our previous results (Fig. 3). To confirm this observation, additional assembly using Unicycler (Wick *et al.* 2017) and Raven (Vaser and Šikić 2021) revealed that the molecule is most likely 74,407 Kbp. We find the same 14 protein-coding mitochondrial genes that were annotated previously (de Melo Teixeira *et al.* 2021) as part of the ubiquinone oxidoreductase, cytochrome b, cytochrome oxidase, and ATP synthase protein complexes. Small and large ribosomal RNAs (*rns* and *rnl*), the RNAseP subunit (rnpB), as well as 24 tRNAs were also annotated.

### Additional minor contigs

Last, 3 remaining scaffolds (7–9) are less than 277 Kbp total, but scaffolds 7 and 8 do contain coding sequences that were not found in the 5 chromosome or mitochondrial DNA assemblies. Inspecting mapping results at the loci on these large scaffolds that were syntenic to the short ones (i.e. the putative duplications), we observed coverage equivalent to the average coverage across the genome. To explore the hypothesis that the small scaffolds were assembly artifacts, we first noted that a large contiguous region on each small scaffold was also present on a large one. We reasoned that, for a given locus in such a region, if it represented a true duplication, short-read sequencing data mapped to a reference containing just one of these 2 putative copies of a given locus would reveal 2-fold higher coverage at the respective site, relative to the genomic average. This was not observed, thus we inferred that the short scaffolds are most likely assembly artifacts, not true duplications, although they remain in the deposited assembly. However, at least some portions of these small contigs contain real genes that fail to assemble into the larger chromosomes. Until additional sequences are completed from other isolates, we propose that these are misassembled scaffolds from 1 of the 5 main chromosomes.

### Gene models

Several metrics suggest a substantial improvement for the gene models of the Silveira genome. We have identified 8,491 total gene models in the new Silveira assembly, which is lower than the previous draft version of this genome, which had 10,228 gene models. The number of exons, mean exon length, and overlapping genes are lower in the new Silveira assembly than the previous one (Table 2). A more fragmented assembly would create discontinuous exons, and thus increase the number of gene models. We have found 207 genes with at least 1 isoform, suggesting alternative splicing. According to the GO enrichment analysis, these genes are related to ribosomal activity. These genes participate in crucial biological processes such as translation, protein modification, catabolism, reproduction, and metabolic processes involving nitrogen and small molecules. The GO enrichment analysis also suggests that those isoforms also play a role as GTPase, kinase, protein/rRNA binding activity, structural molecule and enzyme regulator activity (Supplementary Fig. 2). Finally, the multiple training steps using mRNA sequence data and proteomics, along with the more sophisticated gene predictors implemented in the funannotate pipeline has increased the overall confidence of gene models.

### Secondary metabolism

Previous genomic analysis identified genes associated with secondary metabolite (SM) production that are shared between the 2 *Coccidioides* species, and have experienced positive selection (Sharpton *et al.* 2009). This observed evolutionary pattern might help the organism survive the harsh microenvironments in desert areas where the organism lives, or these SMs may be important for host-pathogen interactions (Perrin *et al.* 2007; Narra *et al.* 2016). Previous analysis suggested that *C. immitis* and *C. posadasii* have 22 and 21 SM clusters (Shang *et al.* 2016) although strain information was not given. We retrieved 23 SM gene clusters using antiSMASH analysis on our new Silveira genome assembly. The biosynthetic class polyketide synthase (PKS) is overrepresented in *Coccidioides* and these sequences display similarity with other well-known PKS clusters in the Ascomycota such as chaetoviridin E, depudecin, nidulanin A, cichorine, shanorellin, aflatoxin, stipitatic acid, and leucinostatin A. The genomes of closely related dermatophyte species also contain 23–25 SM clusters, but only 9 of these clusters are shared between *Coccidioides* and the dermatophytes, which might be related to the diverse ecological niches occupied by the 2 groups of Onygenalean fungi (Martinez *et al.* 2012).

### Phylogenomic characterization

Previous studies analyzing the genetic background of the Silveira strains based on microsatellite markers suggested that this strain belonged to the *C. posadasii* Arizona population (Teixeira and Barker 2016). With the advances of genome-based typing methods in *C.* *posadasii*, novel phylogenetic groups were defined within this species as follows: ARIZONA, Clade AZ1, Clade Col. Springs/GT162, TX/MX/SA, and CARIBE (Teixeira *et al.* 2019). By adding Silveira as a reference genome for whole genome typing we confirmed that this strain grouped within *C. posadasii*, ARIZONA clade based on the unrooted phylogenetic tree (Supplementary Fig. 3a). The basal branch of the ARIZONA group (including the Silveira strain) is supported by 99.8% bootstrap and 53% SH-aLRT support in this unrooted tree. To confirm population placement, we observed that the Silveira strain belongs to the ARIZONA population and no admixture was found using fastSTRUCTURE methods (Supplementary Fig. 3b).

## Data availability

*Coccidioides posadasii* strain Silveira used in this study is available from BEI Resources (NR-48944). This strain has been deposited by other researchers previously to ATCC and Westerdijk Institute/CBS-KNAW resources (ATCC 28868 and CBS 113859). The first Sanger-based genome assembly of *C.* *posadasii* strain Silveira is at NCBI (https://www.ncbi.nlm.nih.gov/nuccore/294654294) In addition, the *C.* *immitis* strains RS and WA_211 genome data were retrieved from NCBI (https://www.ncbi.nlm.nih.gov/nuccore/AAEC00000000 v3, and https://www.ncbi.nlm.nih.gov/nuccore/1695747985). Sequence data and the final assembly generated in this manuscript were submitted to GenBank under BioProject PRJNA494320 (https://www.ncbi.nlm.nih.gov/bioproject/494320). PacBio Sequel sequencing data are found at SRR9644375. Illumina MiSeq sequencing data are found at SRR9644374. Final nuclear assembly is deposited as accession CP075068-CP075075 and the mtDNA genome as accession CP075680.

Supplemental material is available at *G3* online.

## Supplementary Material

jkac031_Supplemental_Figure_1

jkac031_Supplemental_Figure_2

jkac031_Supplemental_Figure_3

jkac031_Supplemental_Table_1
