# Supplementary material for: A chromosomal-level reference genome of the widely utilized Coccidioides posadasii laboratory strain “Silveira”
Source: G3 (Bethesda). 2022 Feb 7;12(4):jkac031. doi: 10.1093/g3journal/jkac031 (PMC8982387; doi:10.1093/g3journal/jkac031)
Supplement: jkac031_Supplemental_Figure_1 [file jkac031_supplemental_figure_1.pdf]

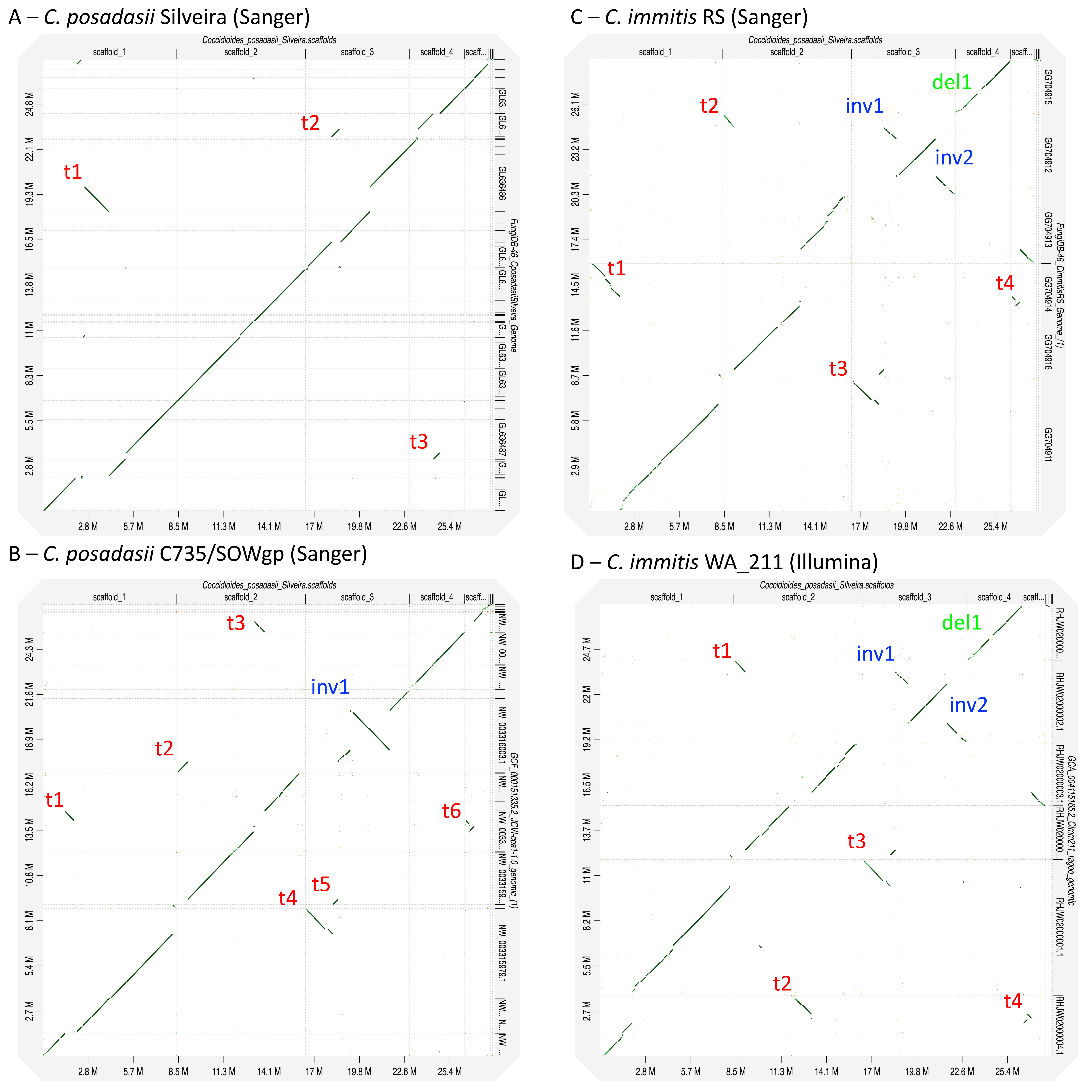
A – C. posadasii Silveira (Sanger)
Coccidioides_posadasii_Silveira.scaffolds
scaffold_1
scaffold_2
scaffold_3
scaffold_4
scaff...
FungiDB-46_CposadasiiSilveira_Genome
t1
t2
t3
2.8 M
5.7 M
8.5 M
11.3 M
14.1 M
17 M
19.8 M
22.6 M
25.4 M
C – C. immitis RS (Sanger)
Coccidioides_posadasii_Silveira.scaffolds
FungiDB-46_CimmitisRS_Genome_(1)
GG704915
GG704912
GG704913
GG704914
GG704916
GG704911
del1
inv1
inv2
t1
t2
t3
t4
B – C. posadasii C735/SOWgp (Sanger)
Coccidioides_posadasii_Silveira.scaffolds
GCF_000151335.2_JCVI-cpa1-1.0_genomic_(1)
inv1
t1
t2
t3
t4
t5
t6
D – C. immitis WA_211 (Illumina)
Coccidioides_posadasii_Silveira.scaffolds
GCA_004115165.2_Cimm211_ragoo_genomic
del1
inv1
inv2
t1
t2
t3
t4
