# Supplementary figures and images for: A chromosomal-level reference genome of the widely utilized Coccidioides posadasii laboratory strain “Silveira”
Source: G3 (Bethesda). 2022 Feb 7;12(4):jkac031. doi: 10.1093/g3journal/jkac031 (PMC8982387; doi:10.1093/g3journal/jkac031)

## A Biological process

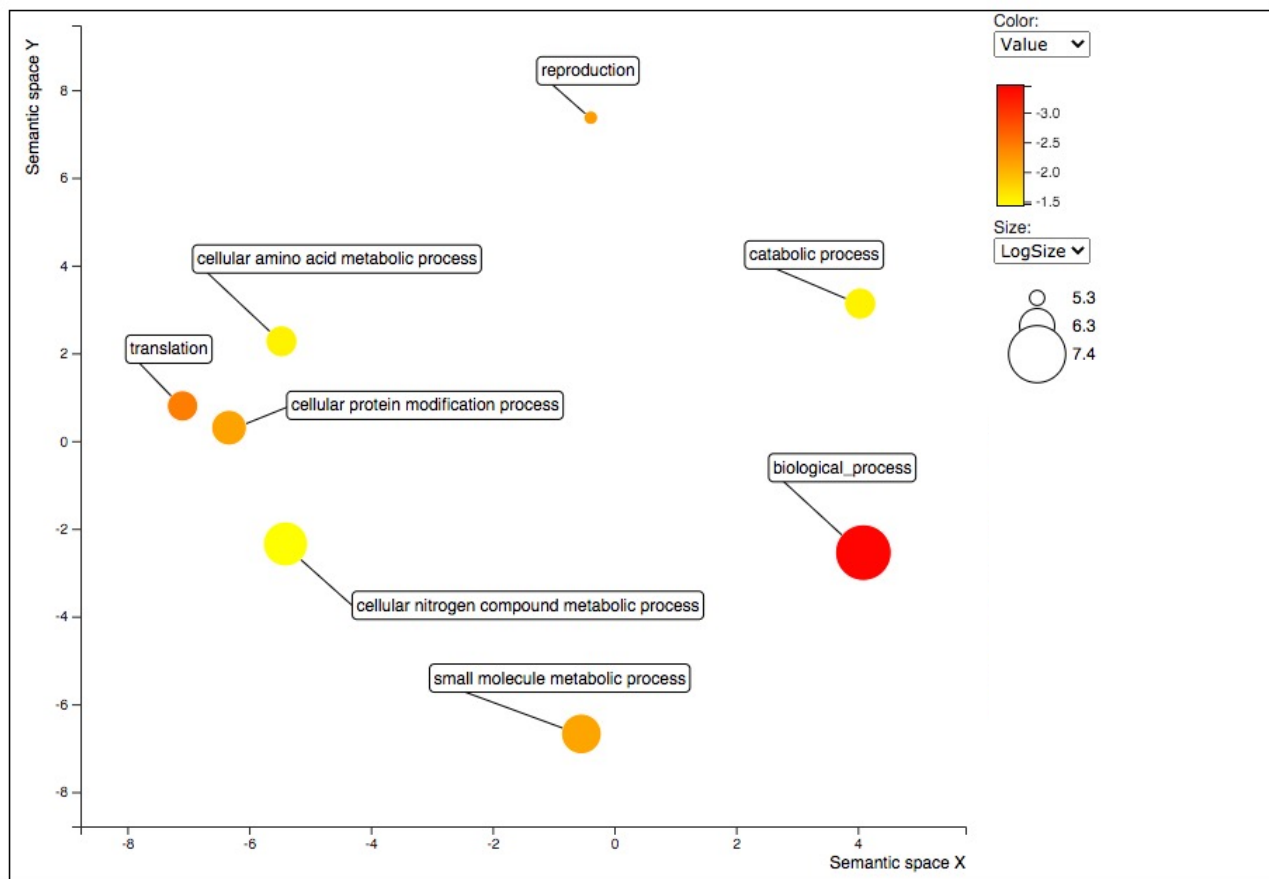

## B Molecular Function

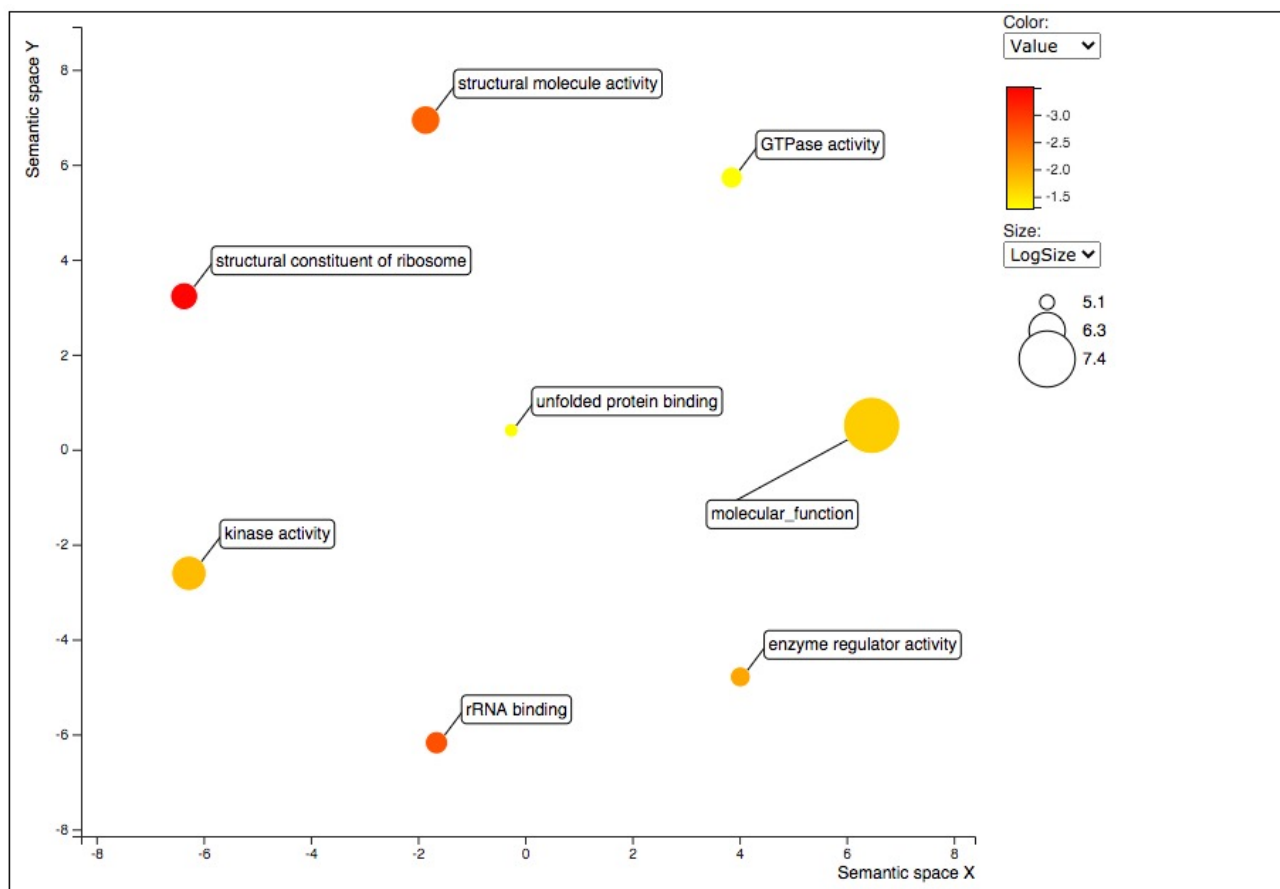

Supplement: jkac031_Supplemental_Figure_2 [file jkac031_supplemental_figure_2.pdf]

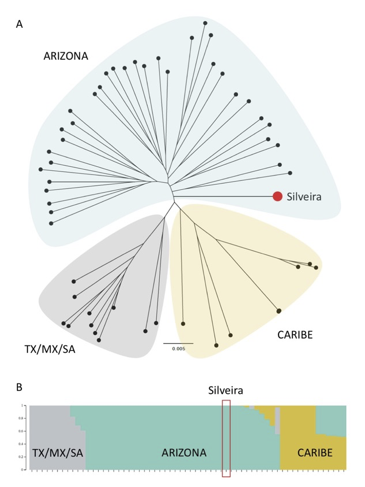

Supplement: jkac031_Supplemental_Figure_3 [file jkac031_supplemental_figure_3.zip › jkac031_Supplemental_Figure_3.tif]
